# Dosimetric Application of Phosphorus Doped Fibre for X-ray and Proton Therapy

**DOI:** 10.3390/s21155157

**Published:** 2021-07-30

**Authors:** Olugbenga J. Olusoji, Crystal Penner, Camille Bélanger-Champagne, Wern Kam, Michael Martyn, Peter Woulfe, Cornelia Hoehr, Sinead O’Keeffe

**Affiliations:** 1 Optical Fibre Sensors Research Centre, University of Limerick, V94 T9PX Limerick, Ireland; wern.kam@ul.ie (W.K.); Peter.Woulfe@galwayclinic.com (P.W.); sinead.okeeffe@ul.ie (S.O.); 2 TRIUMF, 4004 Wesbrook Mall, Vancouver, BC V6T2A3, Canada; cbchampagne@triumf.ca (C.B.-C.); choehr@triumf.ca (C.H.); 3 Health Research Institute, University of Limerick, V94 T9PX Limerick, Ireland; 4 Department of Radiotherapy Physics, Galway Clinic, H91 HHT0 Galway, Ireland; Michael.Martyn@galwayclinic.com

**Keywords:** fibre sensors, optic fibres, radiation induced attenuation, dosimetry, radiation monitoring, phosphorus, proton, X-ray

## Abstract

Phosphorous-doped silica optical fibres with a core diameter of 4 µm were tested in X-ray and proton fields for application in cancer therapy dosimetry. Specifically, the radiation-induced attenuation was investigated in terms of linearity in deposited dose in 15 MV and 6 MV photons and 74 MeV protons, as well as Bragg-peak detection along the proton track. Fibres were found to demonstrate linear relative dose response in both radiation modalities, but possible saturation did occur at the high linear energy transfer of the Bragg peak. This demonstrates the possibility to use these fibres as a relative dosimeter for radiation therapy applications.

## 1. Introduction

As cancer is one of the leading causes of death in developed countries, more and more cancer patients are receiving radiation therapy. Radiotherapy, either alone or in combination with other treatment modalities, requires delivery of the prescribed dose safely and effectively with minimal safety margins, which co-irradiate healthy tissue. Advancement in absolute and relative dosimetry hinges on novel detector solutions to measure the delivered dose in real-time with sufficient spatial resolution for steep radiation field fall-offs and small treatment fields. A dosimeter should be independent of dose rate, beam energy and linear in dose. Ideally, it should also be unaffected by large temperature changes and the presence of an electromagnetic field. The possibility to measure the dose in-vivo during the treatment will allow the direct monitoring of the treatment and intervention in real-time should the delivered dose field significantly differ from the prescribed, further improving treatment outcome and possibly enabling the delivery of larger doses to the tumour. All these aspects could significantly contribute to increasing the therapeutic index of the treatment.

To date, several materials have been explored and various mechanisms have been used for real-time in-vivo dosimetry in which optical fibres (OFs) have played a promising role. The interaction of ionizing radiation with different OF materials differs depending on the microscopic nature of the material and extensive research has been conducted to associate the structural properties of OFs to the response during radiation [1]. In the past, special interest was placed on radiation hardened OFs because of their application in fibre amplifiers and fibre systems for communication in space systems [2,3,4,5].

Ionizing radiation interacts with silica material to generate radiation induced attenuation (RIA), radiation induced emission (RIE), or radiation induced refractive index change (RIRIC). RIRIC is the generation of a point defect from the absorption of ionizing radiation and can be described by Kramer’s-Kronig relation [6]; RIE is the radioluminescence generated in the fibre itself and is made up of fluorescence, phosphorescence and Cerenkov emissions [7,8].

RIA is the loss in the transmission of light signal in the fibre from the degradation of the fibre due to the formation of colour centres in the silica network. These colour centres are formed from the release of electrons leaving holes trapped as defects in the silica network to form other networks, or by the dissociation of electrons and holes from their network to form new defect sites [9]. The formation of these colour centres is dependent on the radiation dose, dose rate, the type of radiation, the dopant in the material, manufacturing conditions and ambient temperature during irradiation [10].

The presence of phosphorus in an OF optimises the refractive index profile and is often used to modify the viscosity of the cladding and core of the OF. Phosphorous is often used together with aluminium to prevent clustering of rare-earth dopants in the silica network and it makes the fibre more sensitive to radiation [10,11,12]. Four distinct intrinsic paramagnetic defect sites P_1_, P_2_, P_3_ and POHC are associated with the presence of phosphorus in a glass material [13]. RIA is the predominant effect from the interaction of phosphorus-doped silica fibres with ionizing radiation and it has been shown that the sensitivity of P-doped silica fibres to ionizing radiation could make them a viable radiation dosimeter for both high and low energy radiation as well as in neutron, X-ray and high-energy proton irradiations [14,15,16,17,18,19,20].

Many studies have shown the viability of doped silica fibres for characterizing proton energy deposition as a function of water depth using radiation-induced luminescence (RIL), with dopants providing luminescent centres and light production being the notable signal source (see e.g., [21]). The primary difficulty in using RIL for proton detection, however, is the accurate expression of the Bragg peak, the area of highest dose deposition (or highest linear energy transfer (LET)) being the most difficult to detect. The difficulty arises from the saturation of scintillating elements causing under response in the Bragg peak region [22,23,24]. This leads to an energy dependence of the OF response in proton fields, an undesirable property which makes clinical implementation difficult. The advantage of RIA in OF is that it is dependent on the signal losses during irradiation and can be extended for distributed sensing using the effect of the RIA on Rayleigh scattering at appropriate wavelengths [25,26].

This study aims to use a 4 µm diameter phosphorus-doped silica fibre to exploit its radiation sensitive properties for relative dosimetry. An initial investigation into the possibility of using the RIA in clinical photon irradiations is presented in this work, along with the possibility of using the RIA to accurately characterize the Bragg peak in a clinical proton beam.

## 2. Materials and Methods

### 2.1. INO Fibre Sample

**Figure 1:**
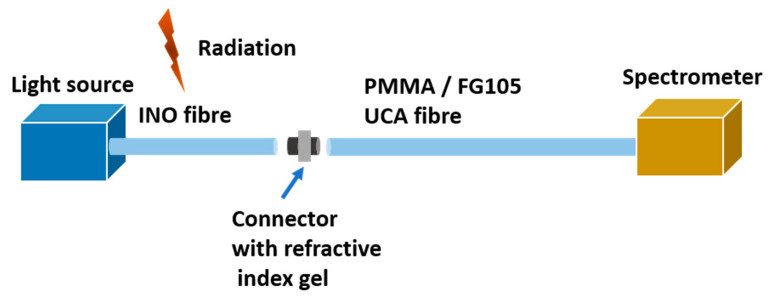
Setup of the experiment.

The fibre tested for RIA was a 3 m long sections of 4 um core diameter phosphorus-doped silica fibre made by Institut National Optique (INO), Quebec, Canada. The extremely fine fibre is further clad to 125 µm and coated to a total diameter of 250 µm. The fibre has an intrinsic attenuation of 5.7 dB/km at 1064 nm and is typically employed in Raman spectroscopy applications. In addition, the fibre was jacketed for further mechanical protection and ambient light elimination using Thorlabs black furcation tubing (FTO20-BK) which has an outer diameter of 2.0 mm. It was terminated at both ends using SMA905 connectors and polished. Separate fibres were used for each energy spectrum in the X-ray beam and a single fibre was used for all positions in the proton beam test. The general setup of the measurement of the RIA from the proton and X-ray irradiation is shown in Figure 1 below.

### 2.2. X-ray Irradiation

**Figure 2:**
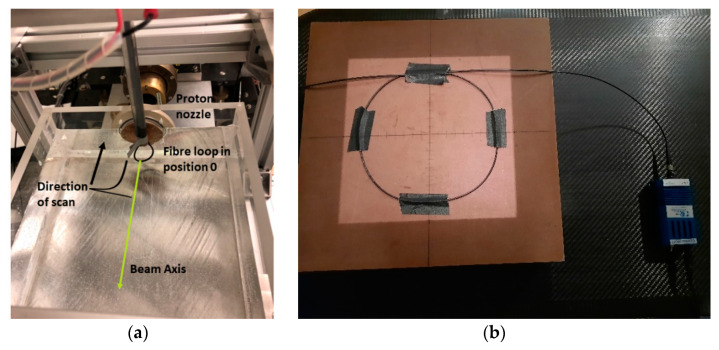
(**a**) setup of the fibre loop in the water box for proton radiation (**b**) setup of the fibre loop on a water equivalent material for X-ray radiation.

The X-ray experiment was conducted at an active clinical facility, the Galway Clinic, Galway, Ireland, with a Siemens Artiste linear accelerator (Linac). The INO OF was placed at a source to surface distance (SSD) of 100 cm and measurements were performed for two clinically relevant X-ray beam energies (6 MV and 15 MV) at a field size of 20 cm × 20 cm with a flattening filter. Fifty cm of the fibre was arranged in a loop, on top of a 10 cm thick water equivalent material to ensure full back-scattering conditions, as shown in Figure 2b. One end of the fibre was connected to an Ocean Optics LS-1 series Tungsten-Halogen light source (wavelength range 360–2000 nm) and the other to an extension OF composed of a 100 µm core custom glass fibre FG105UCA from Thorlabs with an excellent transmission between 200 nm to 1200 nm. The 20 m extension transmitted light from the source to an Ocean optics HDX spectrometer.

Six MV radiation was delivered at a rate of 300 MU/min (monitor units/min) with a total of 1000 MU delivered to the fibre at room temperature; 15 MV radiation was delivered at a rate of 500 MU/min to a total of 1000 MU: at depth dmax, 1 MU corresponds to 1 cGy. Dmax occurs at different depths in water for different energies at a distance 100 cm from the source, The dose delivered was estimated using a clinical treatment planning system on the surface of the water phantom: 3.7 Gy at 6 MV and 2.2 Gy at 15 MV.

### 2.3. Proton Irradiation

RIA tests were conducted at the TRIUMF Proton Therapy Research Facility on the 74 MeV beam from beamline 2C1, the beamline previously used for clinical treatment of ocular melanomas at TRIUMF (1995–2019) [27]. The beam was collimated to 25 mm diameter at the end of the treatment nozzle, and the dose delivered was monitored with a parallel-plate ionization chamber installed upstream of the nozzle, which measures the charge from the ionization in units of monitor counts (MC). The actual conversion into dose is dependent on the energy of the proton beam at each measurement position.

One end of the INO OF was coupled to an ocean insight tungsten-halogen light source (HL-2000-LL) and the other end was coupled to a 15 m length of 1 mm diameter poly-methyl-methacrylate (PMMA) OF to transport the light from the shielded irradiation area to the control room. In the control room, the signal was recorded by a spectrometer (Spectral Products SN OM0P5439-EU) with an integration time of 250 μs for the duration of the irradiation, which lasted typically around 3.05 min.

A portion of the 3 m P-doped OF was fixed in a drop-shaped loop, where 5 cm of fibre would be irradiated; the loop was attached to an acrylic rod which was stepped axially through a water phantom set in a 3-D scanning stage (Figure 2a). The OF was aligned laterally with a laser. The water phantom has a 1 mm thick solid-water entrance window. Irradiations were carried out at several positions after the window from a maximum depth at the Bragg peak (the area of highest LET) to the entrance (36.3, 34.8, 32.4, 30.0, 27.7, 23.7, 15.8, 7.9 and 0 mm) the fibre was irradiated with a beam current of 6 nA for a duration of 400,000 MC each. At the Bragg peak, the conversion from MC to dose is ~140 cGy/10,000 MC. We estimate that due to the fine size of the fibre, that position 36.5 mm and 34.9 mm straddled the actual Bragg peak. The doses at the specific positions have been scaled from the maximum at the Bragg peak according to the dose deposition as measured with a Marcus chamber (a parallel-plate ionization chamber that is widely used for proton therapy dosimetry). After the initial progression from the Bragg peak to the phantom entrance window, the fibre was returned to position 36.5 mm for single, final irradiation for comparison to the first Bragg peak measurement.

### 2.4. Data Analysis

The standard relation used to compute the RIA is the modified Beer-Lambert law of absorption which shows the direct relationship between the attenuation coefficient and power losses per unit length (Equation (1)). Taken into consideration are the change in intensity at specific wavelength at specific time and the length of the fibre: this equation is a good representation of the overall changes in the fibre without considering other microscopic changes.

(1) $$\mathrm{RIA}\left(\mathrm{dB}\right)=-\frac{10}{L}log\left\{\frac{I_{T}\left(\lambda ,t\right)}{I_{i}\left(\lambda \right)}\right\}$$

where L is the length of the irradiated fibre, $I_{T}\left(\lambda ,t\right)$ is the intensity count measured at end of the irradiated fibre during the irradiation and $I_{i}\left(\lambda \right)$ is the initial intensity count measured just before the fibre was irradiated.

## 3. Results

### 3.1. X-ray RIA Results—6 MV

**Figure 3:**
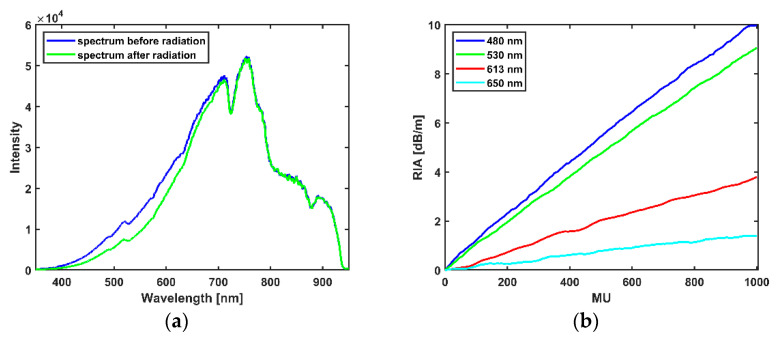
(**a**) Wavelength spectrum before and after radiation. (**b**) Dependence of the RIA on MU for 6 MV X-ray radiation.

In 6 MV X-rays, the observed trend in RIA in the INO OF is a linear increase with dose within the range of the delivered dose: the fibre showed a linear behaviour at all the selected wavelengths as shown in Figure 3b. Wavelengths selected are points along the spectrum with good signal to noise ratio. The sensitivity (linear regression of the RIA with dose) of the selected wavelength increases as the wavelength tends towards the UV as shown in Figure 3b with values as high as 10.10 × $10^{-3}$ dB/m/MU at 480 nm and as low as 1.48 × $10^{-3}$ dB/m/MU at 650 nm as shown in Table 1 below. The spectrum of the transmitted light through the OF before and after the X-ray radiation is shown in Figure 3a while the increase of RIA with increasing MU is shown in Figure 3b, the RIA was smoothened with a Savitzky-Golay filter [28] with a polynomial order of 1 and a frame length of 9.

### 3.2. X-ray RIA Results—15 MV

**Figure 4:**
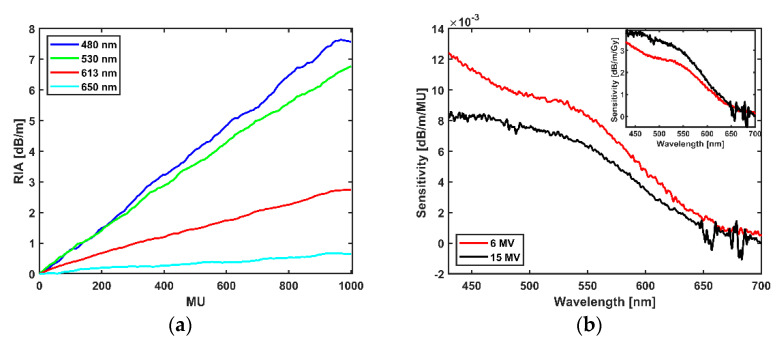
(**a**) Dependence of the RIA on dose (MU) for 15 MV X-ray radiation. (**b**) Sensitivity of the fibre across wavelengths for 6 MV and 15 MV radiation for 1000 MU with inset showing the graph of the conversion to the estimated dose delivered at 100 cm from the source.

Figure 4a shows the RIA of the INO OF with dose from the irradiation of the OF with flattened filtered X-ray beam of 15 MV energy to a total dose of 1000 MU (2.2 Gy); for the selected values as shown in Table 2, sensitivity is as high as 8.08 × $10^{-3}$ dB/m/MU at 480 nm and as low as 0.061 × $10^{-3}$ dB/m/MU at 650 nm. The sensitivities across all the exposed wavelengths for both 6 MV and 15 MV in dB/m/MU with an inset of the conversion to dB/m/Gy are shown in Figure 4b.

### 3.3. Proton RIA Results

**Figure 5:**
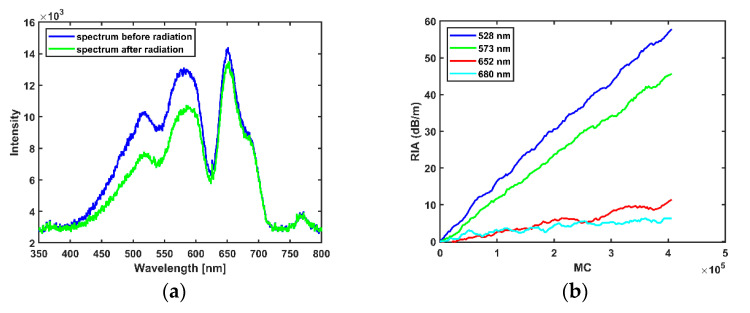
(**a**) Wavelength spectrum before and after radiation in the Bragg peak. (**b**) Dependence of the RIA on MC.

Figure 5a shows the initial spectrum within the visible wavelength measured by the spectrometer before the commencement of proton irradiation, and the spectrum measured at the end of the first proton irradiation at the first Bragg peak position. The intensity difference before and after the irradiation is more pronounced in the wavelength range of 400 nm to 600 nm. The intensity spectrum in Figure 5a differs from the intensity spectrum of the X-ray beam in Figure 3a due to different light sources and extension fibres: in proton measurements, a PMMA fibre was used, while in X-ray measurements a FG 105 UCA (silica) fibre was used, the differing transmission fibre materials have different intrinsic attenuation. The difference in the extension fibre does not affect RIA measurement, however: RIA is dependent on the intensity before irradiation and the change in intensity during irradiation as stated in Equation (1).

Using Equation (1) to calculate the change in intensity, the growth of the RIA in the fibre is shown to be linear. The slopes of the fit lines for selected wavelengths, (see Figure 5b) indicate sensitivities at selected wavelengths as shown in Table 3 below, the RIA was smoothened with a Savitzky-Golay filter [28] with a polynomial order of 1 and a frame length of 9.

**Figure 6:**
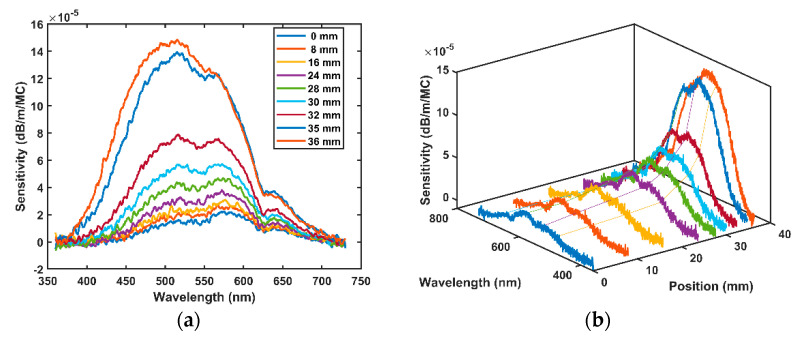
(**a**) 2D view of fibre sensitivity with wavelength at various positions along the proton track; (**b**) 3D view of fibre sensitivity with wavelength at different water depths showing the typical shape of the Bragg peak.

After the initial irradiation in the Bragg peak, the fibre was then stepped back from the Bragg peak to the entrance (position 0) of the water phantom and the RIA was measured at each position. The overall RIA across all wavelengths in the fibre reduces in magnitude as the fibre moves towards the entrance, see Figure 6a. Again, the sensitivities across all wavelengths were computed by fitting a linear fit across all wavelengths with slope representing the sensitivity as shown in Figure 5b. The sensitivity is highest at the Bragg peak and decreases as the fibre is positioned closer and closer to the entrance window (see Figure 6b) indicating a strong dependence on energy with a larger RIA in the area of higher LET, as expected. The sensitivity is expressed in dB/m/MC as it is the metric that unites the duration of irradiation at each position: the dose delivered per MC differs at each position as shown in Table 4. The dose at the Bragg peak is well known, and estimates at other positions are scaled as per characterization by the Markus chamber.

**Figure 7:**
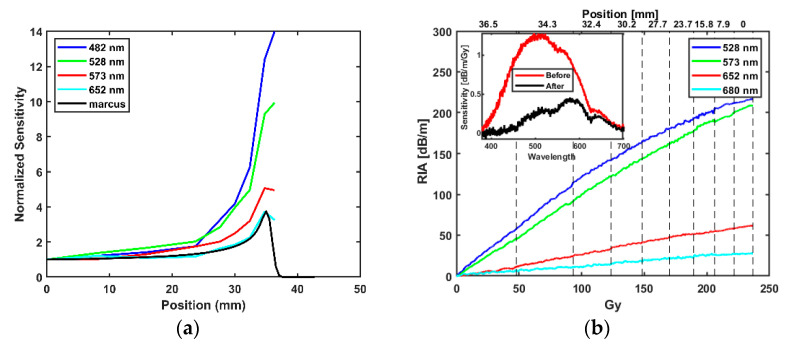
(**a**) Bragg peak detected in the water phantom by the RIA as measured with the INO OF at selected wavelengths. (**b**) RIA from accumulation of dose converted to Gy from Table 4 at selected wavelength where grid lines (position (mm)) indicate estimated delivered dose at that position; inset graph shows the comparison of sensitivity measured at 36.5 mm at the start of all proton irradiation (red) and afterwards (black).

Figure 7a shows the evolution of sensitivity dependence as the fibre was moved along the proton track; the sensitivity was normalized to the sensitivity at the entrance dose i.e., position 0 mm, and compared to the Markus chamber measurement of the energy deposition. To determine if an over or under-response is occurring in a sensor in the high LET area of the Bragg peak, the peak signal is typically compared to the entrance signal and the ratio between the two is compared to the Markus chamber standard. In the case of the INO OF, the peak-to-entrance ratio is wavelength dependent with roughly 14 at 480 nm down to 3.8 at 652 nm. The Markus chamber peak-to-entrance ratio at the TRIUMF proton therapy research facility is 3.8.

Figure 7b shows the accumulation of RIA at selected wavelengths at all positions measured with an inset showing the initial Bragg peak measurement at position 36.5 mm as well as the single final measurement at the same position. A saturation effect due to the deposited dose over the course of the measurements is observed.

## 4. Discussion

### 4.1. Dose Dependence

An important criterion for a dosimeter is the linear increase in measured dose as the delivered dose is increased. At both X-ray energies, the RIA was found to increase linearly with dose. A linear increase with dose was also found for each position along the beam track for proton irradiations.

The linear behaviour recorded for the proton and both photon beams is in accordance with previous reports on the linear behaviours of phosphorus-doped fibres with dose [15,29]. Results from previously reported electron spin resonance (ESR) and absorption measurement of phosphorus-doped glass stated that the high sensitivity of the fibre can be associated with the generation of two forms of phosphorus-oxygen hole centres which are both metastable and stable in the visible wavelengths [1,13,29,30].

### 4.2. Energy Dependence

The INO OF shows an energy dependence in the high (LET) region of the Bragg peak for protons as well as between the 6 and 15 MV X-ray beams. The sensitivities measured for the fibre at 6 MV and 15 MV in dB/m/MU differs (Figure 4b), where 6 MV indicates a higher sensitivity based on MU delivered which is to be expected based on relative depths of dmax in clinical photon beams [31]. A reversal in the sensitivity comparison was observed after conversion from MU to dose in Gy (dB/m/Gy) (Figure 4b inset or Table 1 and Table 2). At the photon energies considered in this work, Compton scattering is the dominant interaction process. For Compton interactions, as the photon energy increases, so too does the proportion of the incoming photon energy transferred to the Compton electrons, resulting in greater energy absorption in the fibre material [32]. This characteristic of the Compton interaction process may go some way towards explaining the results presented in this work (i.e., the greater observed sensitivity, in dB/m/Gy, for 15 MV compared to 6 MV).

In the proton beam the large variation in the relative sensitivity at different wavelengths in the Bragg peak position is higher (Figure 7a) because the production of defect sites of OFs tends to saturate or “quench”. In this experiment, a deviation from linearity in RIA was observed as the INO OF moved towards the entrance of the water phantom. This could possibly be due to accumulated depletion of the defect sites over the course of the previous irradiations. While the saturation effect of the depletion occurred at all wavelengths, as wavelength increased, the overall saturation effect was lower because of the lower sensitivity (i.e., lower RIA) (Figure 7b). This explains why the discrepancy between the Markus chamber peak-to-entrance ratio and the higher wavelength of 653 nm was smaller (Figure 7a) and is consistent with the depletion hypothesis, as the depletion of the colour centre across all positions is lower at this wavelength, see Figure 7b.

## 5. Conclusions

In this work, the effect of low doses of X-ray and proton radiation on phosphorus-doped fibres from INO was investigated for possible use in dosimetry applications. The study indicates the radiation-induced alteration in glass properties results in RIA for all different particles and energies tested as expected based on known interactions of particles with matter [33,34,35,36]. The RIA in the fibre for both the proton and X-ray beam is linear within the dose delivered. Overresponse of the fibre in the Bragg peak of the proton beam suggests the fibre is too sensitive for long irradiations at high LET, especially for wavelengths with higher sensitivity because of the saturation effect; wavelengths with lower sensitivity are better suited for dosimetry applications. These fibres show promise for low dose applications and merit further study at such doses.

## Figures and Tables

**Table 1:** Sensitivity of INO fibre at selected wavelengths for fibre irradiated with X-ray beam energy of 6 MV (dB/m/MU) and the estimated dose at 6 MV (dB/m/Gy) spectra.

Wavelength (nm)	$\mathrm{Sensitivity}\;\mathrm{at}\;6\;\mathrm{MV}\;(\mathrm{dB}/m/\mathrm{MU})\times 10^{-3}$	Sensitivity at 6 MV (dB/m/Gy)
480	10.10	2.7563
530	9.06	2.4725
613	3.87	1.0559
650	1.48	0.4044

**Table 2:** Sensitivity of INO fibre at selected wavelengths for fibre irradiated with X-ray beam energy of 15 MV and the estimated dose at 15 MV spectra.

Wavelength (nm)	$\mathrm{Sensitivity}\;\mathrm{at}\;15\;\mathrm{MV}\;(\mathrm{dB}/m/\mathrm{MU})\times 10^{-3}$	Sensitivity at 15 MV (dB/m/Gy)
480	8.08	3.6945
530	6.88	3.1422
613	2.72	1.2441
650	0.61	0.2793

**Table 3:** Sensitivity of INO fibre at Bragg wavelength for fibre irradiated with proton beam.

Wavelength(nm)	$\mathrm{Sensitivity}\;\mathrm{at}\;\mathrm{Bragg}\;\mathrm{Peak}\;(\mathrm{dB}/m/\mathrm{MC})\times 10^{-4}$	Sensitivity at Bragg Peak (dB/m/Gy)
528	1.4166	1.2011
573	1.1491	0.9842
652	0.2741	0.2324
680	0.1241	0.1053

**Table 4:** Estimated dose conversion for the proton spectra for 10,000 MC to cGy at each position (characterization from the Markus chamber).

**Position (mm)**	36.3	34.8	32.4	30.0	27.7	23.7	15.8	7.9	0
**Dose (cGy/10,000 MC)**	117.9	113.3	75.1	62.0	54.8	47.9	41.8	39.0	37.4

## Data Availability

Not applicable.

## References

[1] Girard S., Alessi A., Richard N., Martin-Samos L., De Michele V., Giacomazzi L., Agnello S., Di Francesca D., Morana A., Winkler B. (2019). Overview of radiation induced point defects in silica-based optical fibers. Rev. Phys..

[2] Shikama T., Kakuta T., Shamoto N., Narui M., Sagawa T. (2000). Behavior of developed radiation-resistant silica-core optical fibers under fission reactor irradiation. Fusion Eng. Des..

[3] Brichard B., Fernandez A.F., Ooms H., Berghmans F., Decréton M., Tomashuk A., Klyamkin S., Zabezhailov M., Nikolin I., Bogatyrjov V. (2004). Radiation-hardening techniques of dedicated optical fibres used in plasma diagnostic systems in ITER. J. Nucl. Mater..

[4] Girard S., Keurinck J., Boukenter A., Meunier J.P., Ouerdane Y., Azaïs B., Charre P., Vié M. (2004). Gamma-rays and pulsed X-ray radiation responses of nitrogen-, germanium-doped and pure silica core optical fibers. Nucl. Instrum. Methods Phys. Res. Sect. B Beam Interact. Mater. Atoms.

[5] Wijnands T.J., De Jonge L.K., Kuhnhenn J., Hoeffgen S.K., Weinand U. Optical absorption in commercial single mode optical fibres for the LHC machine. Proceedings of the Topical Workshop on Electronics for Particle Physics, TWEPP 2007.

[6] Primak W. (1958). Fast Neutron Induced Changes. Phys. Rev..

[7] Marckmann C.J., Aznar M.C., Andersen C.E., Bøtter-Jensen L. (2006). Influence of the stem effect on radioluminescence signals from optical fibre Al2O3:C dosemeters. Radiat. Prot. Dosim..

[8] Veronese I., Cantone M.C., Catalano M., Chiodini N., Fasoli M., Mancosu P., Mones E., Moretti F., Scorsetti M., Vedda A. (2013). Study of the radioluminesence spectra of doped silica optical fibre dosimeters for stem effect removal. J. Phys. D Appl. Phys..

[9] Ladaci A. (2019). Rare Earth Doped Optical Fibers and Amplifiers for Space Applications.

[10] Girard S., Morana A., Ladaci A., Robin T., Mescia L., Bonnefois J.J., Boutillier M., Mekki J., Paveau A., Cadier B. (2018). Recent advances in radiation-hardened fiber-based technologies for space applications. J. Opt..

[11] Ehrt D., Ebeling P., Natura U. (2000). UV transmission and radiation-induced defects in phosphate and fluoride-phosphate glasses. J. Non. Cryst. Solids.

[12] Origlio G., Messina F., Cannas M., Boscaino R., Girard S., Boukenter A., Ouerdane Y. (2009). Optical properties of phosphorus-related point defects in silica fiber preforms. Phys. Rev. B Condens. Matter Mater. Phys..

[13] Griscom D.L., Friebele E.J., Long K.J., Fleming J.W. (1983). Fundamental defect centers in glass: Electron spin resonance and optical absorption studies of irradiated phosphorus-doped silica glass and optical fibers. J. Appl. Phys..

[14] Girard S., Kuhnhenn J., Gusarov A., Brichard B., Van Uffelen M., Ouerdane Y., Boukenter A., Marcandella C. (2013). Radiation effects on silica-based optical fibers: Recent advances and future challenges. IEEE Trans. Nucl. Sci..

[15] Paul M.C., Bohra D., Dhar A., Sen R., Bhatnagar P.K., Dasgupta K. (2009). Radiation response behavior of high phosphorous doped step-index multimode optical fibers under low dose gamma irradiation. J. Non. Cryst. Solids.

[16] Bradley D.A., Zubair H.T., Oresegun A., Louay G.T., Abdul-Rashid H.A., Ung N.M., Alzimami K.S. (2019). Towards the development of doped silica radioluminescence dosimetry. Radiat. Phys. Chem..

[17] Di Francesca D., Girard S., Agnello S., Alessi A., Marcandella C., Paillet P., Richard N., Boukenter A., Ouerdane Y., Gelardi F.M. (2016). Radiation response of ce-codoped germanosilicate and phosphosilicate optical fibers. IEEE Trans. Nucl. Sci..

[18] West R.H. (2002). P-doped optical fibers in dosimetry. Photonics for Space and Radiation Environments II.

[19] Borgermans P., Brichard B., Berghmans F., Decreton M.C., Golant K.M., Thomashuk A.L., Nikolin I.V. (2001). Dosimetry with optical fibres: Results for pure silica, phosphorous and erbium doped samples. Fiber Optic Sensor Technology II.

[20] Zubair H.T., Oresegun A., Rahman A.K.M.M., Ung N.M., Mat Sharif K.A., Zulkifli M.I., Yassin S.Z.M., Maah M.J., Yusoff Z., Abdul-Rashid H.A. (2019). Real-time radiation dosimetry using P-doped silica optical fiber. Meas. J. Int. Meas. Confed..

[21] Hoehr C., Morana A., Duhamel O., Capoen B., Trinczek M., Paillet P., Duzenli C., Bouazaoui M., Bouwmans G., Cassez A. (2019). Novel Gd3+-doped silica-based optical fiber material for dosimetry in proton therapy. Sci. Rep..

[22] Hoehr C., Penner C., O’Keeffe S., Woulfe P., Capoen B., El Hamzaoui H., Bouwmans G., Morana A., Girard S. (2020). Optical Fibers for Dosimetry in External Beam Therapy. Proceedings of the Optical Sensors and Sensing Congress.

[23] Savard N., Potkins D., Beaudry J., Jirasek A., Duzenli C., Hoehr C. (2018). Characteristics of a Ce-Doped silica fiber irradiated by 74 MeV protons. Radiat. Meas..

[24] Veronese I., Cantone M.C., Chiodini N., Coray A., Fasoli M., Lomax A., Mones E., Moretti F., Vedda A. (2010). Feasibility study for the use of cerium-doped silica fibres in proton therapy. Radiat. Meas..

[25] Toccafondo I., Marin Y.E., Guillermain E., Kuhnhenn J., Mekki J., Brugger M., Pasquale F. (2017). Di Distributed Optical Fiber Radiation Sensing in a Mixed-Field Radiation Environment at CERN. J. Light. Technol..

[26] Sabatier C., Rizzolo S., Morana A., Allanche T., Robin T., Cadier B., Paillet P., Gaillardin M., Duhamel O., Marcandella C. (2018). 6-MeV Electron Exposure Effects on OFDR-Based Distributed Fiber-Based Sensors. IEEE Trans. Nucl. Sci..

[27] Blackmore E.W. Operation of the TRIUMF (20-500 MeV) proton irradiation facility. Proceedings of the 2000 IEEE Radiation Effects Data Workshop, Workshop Record, Held in Conjunction with IEEE Nuclear and Space Radiation Effects Conference (Cat. No.00TH8527).

[28] Savitzky A., Golay M.J.E. (1964). Smoothing and Differentiation of Data by Simplified Least Squares Procedures. Anal. Chem..

[29] Girard S., Ouerdane Y., Marcandella C., Boukenter A., Quenard S., Authier N. (2011). Feasibility of radiation dosimetry with phosphorus-doped optical fibers in the ultraviolet and visible domain. J. Non-Cryst. Solids.

[30] Di Francesca D., Li Vecchi G., Girard S., Alessi A., Reghioua I., Boukenter A., Ouerdane Y., Kadi Y., Brugger M. (2018). Radiation-Induced Attenuation in Single-Mode Phosphosilicate Optical Fibers for Radiation Detection. IEEE Trans. Nucl. Sci..

[31] Xhafa B., Mulaj T., Hodolli G., Nafezi G. (2014). Dose Distribution of Photon Beam by Siemens Linear Accelerator. Int. J. Med. Phys. Clin. Eng. Radiat. Oncol..

[32] Khan F.M., Gibbons J.P. (2014). Khan’s the Physics of Radiation Therapy.

[33] Tavernier S. (2010). Interactions of Particles in Matter. Experimental Techniques in Nuclear and Particle Physics.

[34] Dicello J.F. (2007). Absorption Characteristics of Protons and Photons in Tissue. Technol. Cancer Res. Treat..

[35] Verhey L., Blattman H., Deluca P.M., Miller D. (1998). 4. Proton Interactions with Matter. J. Int. Comm. Radiat. Units Meas..

[36] Newhauser W.D., Zhang R. (2015). The physics of proton therapy. Phys. Med. Biol..

